# Treatment of catheter related thrombosis: A systematic review, meta-analysis, and national survey

**DOI:** 10.1016/j.jvsv.2025.102359

**Published:** 2025-11-29

**Authors:** Laurens A. Oomen, Janette van Diest, Felice R.M. Lucas, Jitske Rijpkema, George L. Burchell, Florianne J.L. van Zanten, Kee F. Choi, Marcella C.A. Muller, Angelique M.E. de Man, Alexander P.J. Vlaar, Jarom Heijmans, Bart J. Biemond, Nick van Es, Jasper M. Smit, Pieter R. Tuinman

**Affiliations:** a Department of Intensive Care, Amsterdam University Medical Centers, Amsterdam, the Netherlands; b Medical Library VUMC, Amsterdam University Medical Centers, Amsterdam, the Netherlands; c Department of Clinical Hematology, Amsterdam University Medical Centers, University of Amsterdam, Amsterdam, the Netherlands; d Department of Vascular Medicine, Amsterdam University Medical Centers, Amsterdam, the Netherlands; e Amsterdam Cardiovascular Sciences, Amsterdam University Medical Centers, Amsterdam, the Netherlands

**Keywords:** Catheter-related thrombosis, Central venous catheters, Peripherally inserted central catheters, Direct oral anticoagulants, Low-molecular-weight heparin

## Abstract

**Background:**

Catheter-related thrombosis (CRT) is a known complication of central venous catheters and peripherally inserted central catheters, yet optimal treatment remains uncertain. We conducted a systematic review and national survey to assess current CRT management strategies.

**Methods:**

Following the PRISMA guidelines, we searched three databases through October 2024 for studies on CRT associated with central venous catheters or peripherally inserted central catheters. Meta-analyses and subgroup analyses were performed by anticoagulant type. A national survey among Dutch intensive care and hematology physicians explored current treatment practices.

**Results:**

Of 4123 records screened, 34 observational studies were included, mostly involving patients with cancer. The venous thromboembolism recurrence rate per 100 patient-years was higher in patients with cancer (14.1; 95% confidence interval, 11.4- 17.4; I^2^ = 35.1) vs patients without cancer (2.0; 95% confidence interval, 0.6-6.0; I^2^ = 10.3; *P* = .0002). Recurrence was comparable between direct oral anticoagulants (DOACs) and low-molecular-weight heparin/vitamin K antagonists (LMWH/VKAs), at 11.0 vs 7.6 (*P* = .14). Major bleeding occurred in 10.5 vs 13.1 (*P* = .45), and clinically relevant nonmajor bleeding in 26.2 vs. 22.4 (*P* = .70), for DOACs vs LMWH/VKAs, respectively. All studies were observational, most at high risk of bias. Survey data showed LMWH was preferred for symptomatic CRT (50%), with treatment lasting 8 days to 6 months. In asymptomatic CRT, anticoagulant type and duration were left to physician discretion in 64% of cases.

**Conclusions:**

Treatment with LMWH/VKA or DOACs shows similarly low venous thromboembolism recurrence, although rates are higher in patients with cancer. Bleeding was substantial and comparable across therapies. Evidence is limited by observational bias. Survey data show that LMWH predominates for CRT, with variable duration. Well-designed randomized controlled trials are warranted.

**Article Highlights**

• **Type of Research:** A systematic review, meta-analysis, and survey
• **Key Findings:** Patients with cancer had a higher venous thromboembolism recurrence rate (14.1 vs 2.0 per 100 patient-years). Anticoagulants (direct oral anticoagulants vs low-molecular-weight heparin [LMWH]/vitamin K antagonists) showed similar recurrence rates (11.0 vs. 7.6) and substantial but comparable bleeding risks (major bleeding: 10.5% vs 13.1%; clinically relevant nonmajor bleeding: 26.2% vs 22.4%).
• **Take Home Message:** Both direct oral anticoagulants and LMWH/vitamin K antagonists show similar efficacy for catheter-related thrombosis, but bleeding risks are substantial and comparable across treatments, with no significant difference in major or clinically relevant nonmajor bleeding. LMWH is preferred for symptomatic catheter-related thrombosis, though treatment duration is variable. Well-designed randomized controlled trials are needed to clarify optimal management.

Central venous catheters (CVCs) are indispensable tools in modern medical practice, facilitating the administration of medications, parenteral nutrition, and long-term intravenous access for patients. However, their use is accompanied by a significant risk of complications, with central venous catheter-related thrombosis (CRT) being a prominent concern. CRT is associated with unfavorable clinical outcomes, including complications such as pulmonary embolism (PE) (10%-15%), catheter malfunction (10%-36%), and post-thrombotic syndrome (7%- 46% of cases).^1^, ^2^, ^3^

Previous studies primarily focused on identifying the incidence of and risk factors associated with CRT development. The reported incidence of CRT overall varies between studies, with variability being influenced by factors such as study design, patient populations, catheter type, and diagnostic methods. The annual incidence of symptomatic and asymptomatic CRT ranges from 0.4 to 1.0 cases per 10,000 individuals, with the highest rates observed in critically ill patients and those with active metastatic malignancies. CRT accounts for nearly 70% of upper extremity deep vein thrombosis (UEDVT) cases and approximately 10% of all venous thromboembolisms (VTEs). Patient-related risk factors include malignancy, sepsis, age, immobility, and thrombophilia.

Non-patient-related risk factors for CRT include catheter type (peripherally inserted central catheters [PICCs] carry approximately a three-fold higher risk than CVCs), number of lumens (triple lumen > double lumen > single lumen), catheter site (femoral > subclavian > internal jugular), and the type of medication or parenteral nutrition administered via the CVC. Another component is the number of attempts inserting the catheter required.^1^^,^^3^^,^^4^

Limited progress in understanding the treatment and management of CRT has resulted in considerable variability in clinical guidelines and practice. Knowledge gaps include the optimal type and duration of anticoagulant therapy, best approaches to line management, and the role of fibrinolytics. Additionally, the management of asymptomatic CRT remains unclear, with increasing detection of these cases, largely owing to the growing use of handheld ultrasound examination.^5^^,^^6^ The lack of clarity in existing CRT guidelines often leaves clinicians without specific direction, leading them to rely on guidelines developed for the management of lower DVT instead.^7^, ^8^, ^9^, ^10^, ^11^

The aim of this systematic review was to evaluate the efficacy and safety of treatment options for CRT and compare clinical outcomes. Additionally, a national survey was conducted in intensive care units (ICUs) and hematology departments in the Netherlands to gain insight into the current clinical practice regarding symptomatic and asymptomatic CRT.

## Methods

A systematic search was performed in PubMed, Embase.com, and the Wiley/Cochrane Library through October 31, 2024. The protocol was not prospectively registered in PROSPERO owing to the retrospective integration of a parallel survey component. However, the review was conducted in accordance with PRISMA guidelines (Supplementary Table I, online only), with assistance from a medical information specialist. The full search strategies are provided in Supplementary Table II (online only). To identify additional studies meeting our inclusion criteria, we also reviewed the reference lists of the relevant articles retrieved.

Articles were eligible for inclusion if they involved patients >18 years of age with objectively confirmed CRT in a CVC, defined as a flexible tube inserted into a large vein (eg, internal jugular, subclavian, or femoral veins), including Port-a-caths, PICCs; other central venous access devices (or a continuous venovenous hemofiltration catheter); and reported outcomes for CRT alone, not combined with other forms of UEDVT.

Abstracts, case studies or series, literature reviews, and articles with no available English translation were excluded.

### Data extraction

Screening of articles was done by two independent researchers (J.D. and F.L.), using Rayyan^12^ for initial sorting and categorization. All studies identified by Rayyan were then manually reviewed by the researchers. Data extraction was systematically performed by one researcher (J.D.), with close consultation and verification by a second researcher to ensure consensus and accuracy (P.R.T.). The extracted data encompassed several key domains. Study characteristics included the aim of the study, as well as its design, geographic location, period of inclusion, sample size, and duration of follow-up. Regarding the study population, details were collected on the type and characteristics of patients, including demographic information, cancer or no active cancer diagnosis, and clinical settings. Where possible, symptomatic and asymptomatic cases of CRT were separated, along with the diagnostic methods used to confirm CRT. Intervention details were also recorded, covering the type of therapy administered, treatment dosage, and duration. For outcomes, we accepted the definitions used in the individual studies, both for thrombotic and bleeding-related outcomes. Given that there can be some heterogeneity in the definitions of bleeding, we chose to adopt the definitions used in each study.

The primary outcome was VTE recurrence, with major bleeding and clinically relevant nonmajor bleeding (CRNMB) as secondary outcomes, following the definitions from the studies. Additional secondary outcomes included PE, line removal, and mortality.

### Quality assessment

Included articles were assessed for bias using the Newcastle Ottawa Scale of bias.^13^ It was chosen owing to comparable reliability as the ROBINS-I, ease of reproduction, and lack of randomization in included observational studies.^14^ The quality assessment was conducted individually by two researchers (L.O., J.D., or F.L.). Disagreement was resolved by consensus meeting with a third reviewer (P.R.T.). In cases where a second cohort was absent, the corresponding point was not assigned. However, this did not affect the overall scoring, and the maximum attainable score remained unchanged.

### Statistical analysis

We report the primary and secondary outcomes using narrative synthesis and, when possible, pooled incidence rate estimates. Incidence rates per 100 person-years of recurrent VTE, major bleeding, and CRNMB (with 95% confidence intervals [CIs]) were pooled using a random intercept Poisson regression model within a generalized linear mixed model (GLMM) framework owing to its advantages in handling sparse data, avoiding arbitrary continuity corrections, and appropriately modeling between-study heterogeneity.^7^, ^8^, ^9^, ^10^, ^15^ The Poisson distribution with a log link function was applied to model the event rates, and the random intercept accounted for variability between studies in baseline event rates. Incidence rates were calculated using person-time, defined as the total time each patient was at risk during follow-up. When person-time was not directly reported in the original studies, we estimated it by multiplying the number of patients by the reported mean or median follow-up duration in the respective groups, depending on availability. Subgroup analyses were performed for anticoagulant type (eg, low-molecular-weight heparin [LMWH]/VKA vs direct oral anticoagulants [DOACs]), and whether CRT was related to malignancy (eg, patients with cancer vs patients without cancer). For the subgroup analysis of VTE recurrence by anticoagulant type, we included only studies that^1^ clearly reported the type of therapeutic anticoagulant used (LMWH, VKA, or DOAC), with outcomes stratified by agent where applicable^2^; had a defined follow-up period and treatment period^3^; and provided a clear definition of recurrent VTE, preferably confirmed by imaging. Furthermore, to ensure consistency and comparability of outcomes, we limited our meta-analysis of major bleeding and CRNMB to studies that used standardized and widely accepted definitions as per the International Society on Thrombosis and Haemostasis. Heterogeneity was assessed using the Cochran Q statistic and quantified with the I^2^ statistic, with an I^2^ of <30% indicating low, 30% to 70% moderate, and >70% high heterogeneity. Publication bias was assessed using funnel plots, comparing the logit event rate against the standard error. A sensitivity analysis was conducted by comparing pooled rates with and without outliers, and by assessing the robustness of the GLMM results using the inverse variance weighting with continuity correction (IV+CC) method. Additionally, sensitivity analyses were performed based on device type (PICC-only vs mixed PICC/CVC cohorts). The Hartung-Knapp-Sidik-Jongman method was used to estimate the CIs of the pooled estimates. All analyses were performed in R using the meta and metafor package (R Foundation for Statistical Computing).^11^^,^^16^ For comparisons of survey outcomes between groups (eg, ICU vs hematology department), we used Fisher's exact test for binary variables and the Mann-Whitney *U* test for ordinal variables.

### Survey

A cross-sectional survey of practice regarding the current treatment of CRT in ICUs and hematology departments across the Netherlands was performed. ICUs and hematology departments were chosen because of the frequent use of CVCs. A list of ICUs was compiled from the website of the NICE registry.^17^ In addition, hematology departments from a convenience sample of the same hospitals were a approached as comparison. Both departments were first contacted by phone (Appendix).

## Results

### Systematic review

**Fig 1:**
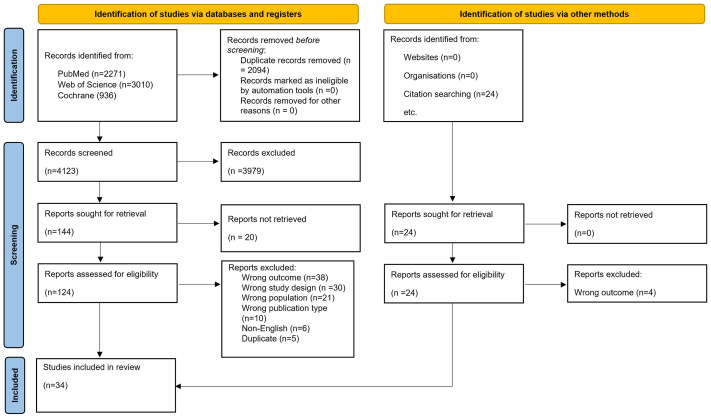
PRISMA flowchart summarizing the identification, screening, and inclusion of studies in the systematic review.

Of 4133 articles identified, full texts of 168 articles were evaluated; 34 met the inclusion criteria (Fig 1).^18^, ^19^, ^20^, ^21^, ^22^, ^23^, ^24^, ^25^, ^26^, ^27^, ^28^, ^29^, ^30^, ^31^, ^32^, ^33^, ^34^, ^35^, ^36^, ^37^, ^38^, ^39^, ^40^, ^41^, ^42^, ^43^, ^44^, ^45^, ^46^, ^47^, ^48^, ^49^, ^50^, ^51^ Of the 34 included observational studies, 10 were prospective and 24 were retrospective in design. No interventional studies were identified.

Most studies focused solely on inpatients or outpatients, with one study exclusively covering critically ill patients with CRT.^36^ However, none of the other studies specified whether critically ill patients were included among the inpatients.

A total of 3764 patients with CRT were included (Supplementary Table III, online only) with a median number of patients per study of 65 (range, 6-663). Data on the number of patients with cancer in the cohorts was reported in 31 studies,^18^^,^^20^, ^21^, ^22^, ^23^, ^24^^,^^26^, ^27^, ^28^, ^29^, ^30^, ^31^, ^32^^,^^34^^,^^35^^,^^37^, ^38^, ^39^, ^40^, ^41^, ^42^, ^43^, ^44^, ^45^, ^46^, ^47^, ^48^, ^49^, ^50^, ^51^ and 4 studies reported outcomes on noncancer-related CRT.^19^^,^^25^^,^^26^^,^^33^ To diagnose CRT, venous duplex ultrasound examination was most frequently used.

Twenty-one studies included only patients with symptomatic CRT(Supplementary Table III, online only).^18^, ^19^, ^20^^,^^22^, ^23^, ^24^^,^^26^, ^27^, ^28^, ^29^, ^30^, ^31^^,^^33^^,^^34^^,^^37^^,^^39^^,^^40^^,^^42^^,^^43^^,^^45^^,^^46^^,^^48^ Three studies included both patients with asymptomatic and symptomatic CRT,^35^^,^^38^^,^^49^ and 10 did not specify the type of CRT (Supplementary Table IV, online only).^21^^,^^25^^,^^32^^,^^41^^,^^43^^,^^44^^,^^46^^,^^47^^,^^50^^,^^51^ No studies reported outcomes only in patients with asymptomatic CRT.

#### Treatments and treatment duration studied

Anticoagulant therapy alone was used to treat CRT in 24 studies, encompassing 2167 patients. Line removal as treatment of CRT with or without anticoagulation was reported in four studies, whereas fibrinolytics with or without anticoagulation were used in six studies (Supplementary Table I, online only).

Among the 24 studies using anticoagulant therapy, six used unfractionated heparin as initial treatment, followed by LMWH and/or a vitamin K antagonist (VKA).^21^^,^^23^^,^^25^^,^^26^^,^^29^^,^^32^^,^^36^^,^^51^ LMWH, whether or not transitioned to long term VKA, was used in 15 studies^24^^,^^29^, ^30^, ^31^^,^^34^^,^^38^, ^39^, ^40^^,^^42^^,^^43^^,^^46^, ^47^, ^48^, ^49^, ^50^ and DOACs in 9 studies.^29^^,^^34^^,^^35^^,^^37^^,^^43^^,^^47^^,^^49^, ^50^, ^51^ Finally, two studies evaluated fondaparinux.^42^^,^^47^ The duration of anticoagulation treatment ranged from 5 days to >6 months.

#### Risk of bias and quality assessment

**Fig 2:**
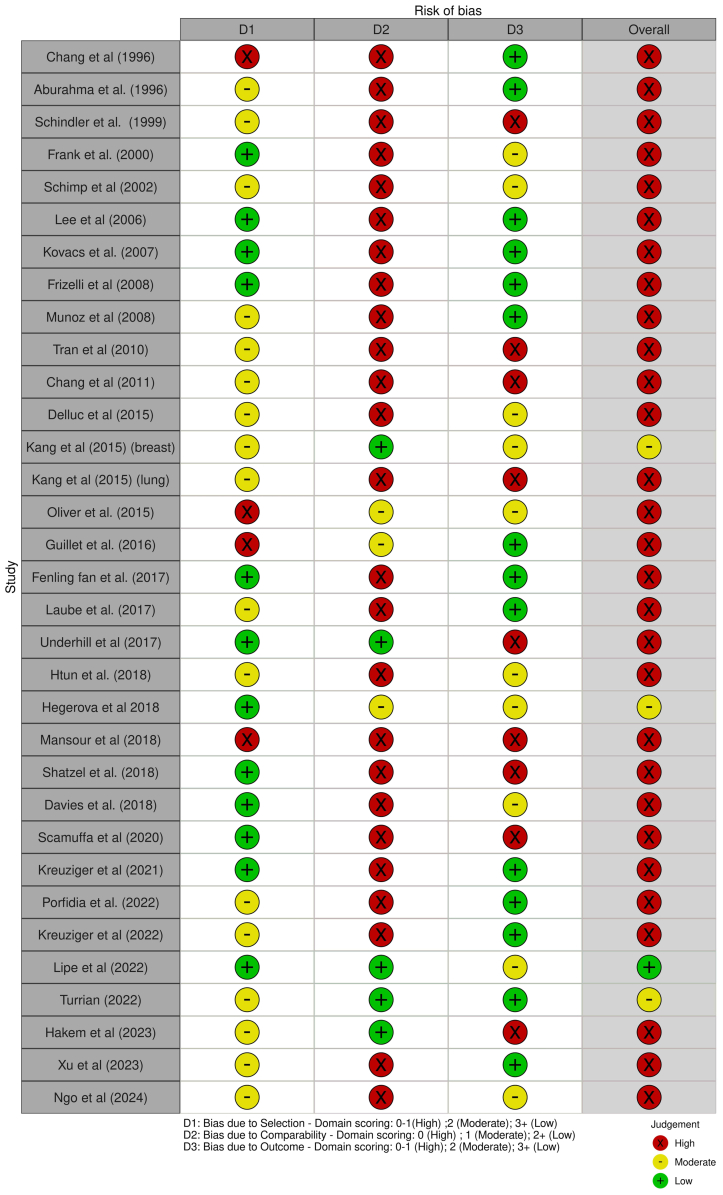
Risk of bias assessment for each study according to Newcastle-Ottawa score.^16^ Plots created using risk-of-bias visualization (ROBVIS) tool.^52^

Study quality was assessed for all included studies (Fig 2). Two studies were rated as high quality (7-9 stars), 3 as moderate quality (5-6 stars), and 29 as low quality (0-4 stars). Notably, studies classified as low quality received poor scores primarily in the comparability or selection domains.

#### Outcomes

Owing to wide variation in outcomes definitions and reporting (Supplementary Table I, online only), 19 studies on anticoagulation were suitable for inclusion in the meta-analysis. Studies assessing line removal, prolonged anticoagulation duration (≥3 months), or the use of fibrinolytics were too heterogeneous and are therefore only presented in the narrative synthesis. Because most eligible studies included only patients with symptomatic CRT or did not specify symptom status, data on symptomatic and asymptomatic VTE were combined.

#### VTE recurrence

**Fig 3:**
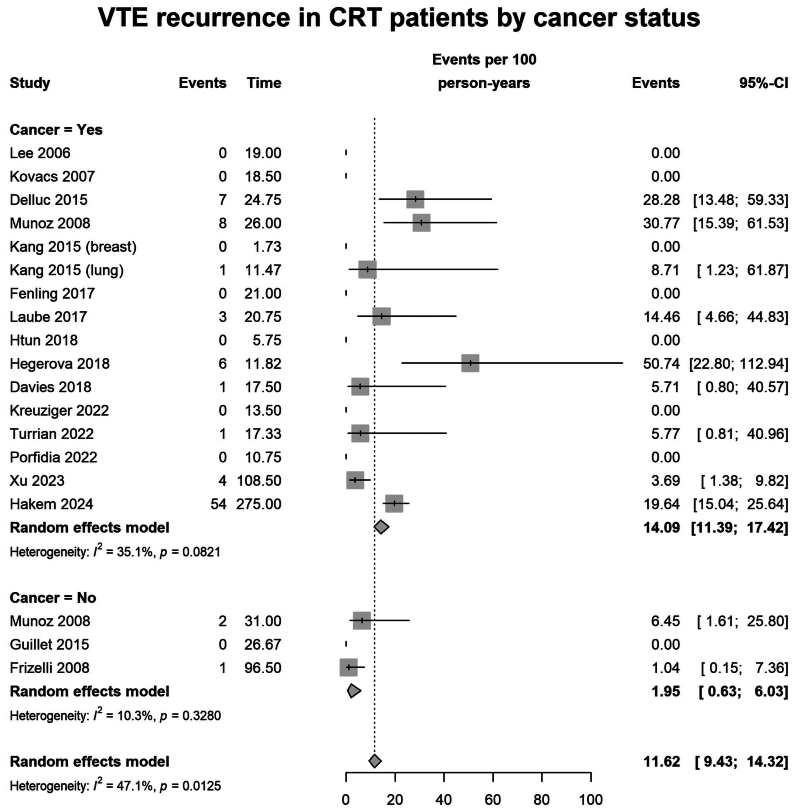
Forest plot of pooled recurrent venous thromboembolism (*VTE*) rate of catheter-related thrombosis (*CRT*) in cancer vs patients without cancer, expressed as events per 100 person-years. *CI*, confidence interval.

With a median follow-up of 3 months (interquartile range, 3.0-13.9 months), the pooled VTE recurrence rate was 11.6 per 100 person-years (95% CI, 9.4-14.3; I^2^ = 47.1%; n = 1762 patients from 19 studies). In the pooled analysis, the duration of follow-up often exceeded the treatment duration, but most studies did not report whether recurrences occurred during or after anticoagulation. Therefore, recurrence rates may reflect both on-treatment and post-treatment events. When stratified by cancer status, the recurrence rate was numerically higher in patients with cancer, with 14.1 per 100 person-years (95% CI, 11.4-17.4; I^2^ = 35.1%; n = 1127 patients across 16 studies), compared with 2.0 per 100 person-years in patients without cancer (95% CI, 0.6-6.0; I^2^ = 10.3%; n = 526 patients across 3 studies; *P* = .0002 for subgroup difference) (Fig 3).

**Supplementary Fig 1:**
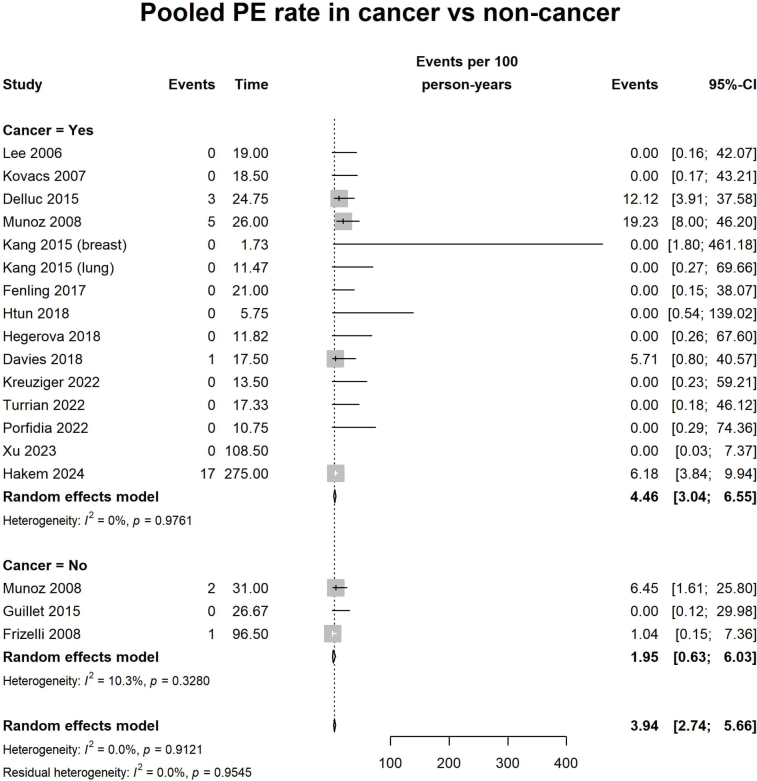
Forest plot of pooled symptomatic pulmonary embolism (*PE*) rate in patients with cancer and catheter-related thrombosis (CRT), expressed as events per 100 person-years. *CI*, confidence interval.

Similarly, PE recurrence rate showed a numerically higher rate in patients with cancer (4.46 per 100 person-years; 95% CI, 3.0-6.6; I^2^ = 0%; n = 1044 patients across 15 studies) compared with patients without cancer (1.95 per 100 person-years; 95% CI, 0.6-6.0; I^2^ = 10.3%; n = 526 patients across 3 studies; *P* = .17 for subgroup difference) (Supplementary Fig 1, online only).

**Fig 4:**
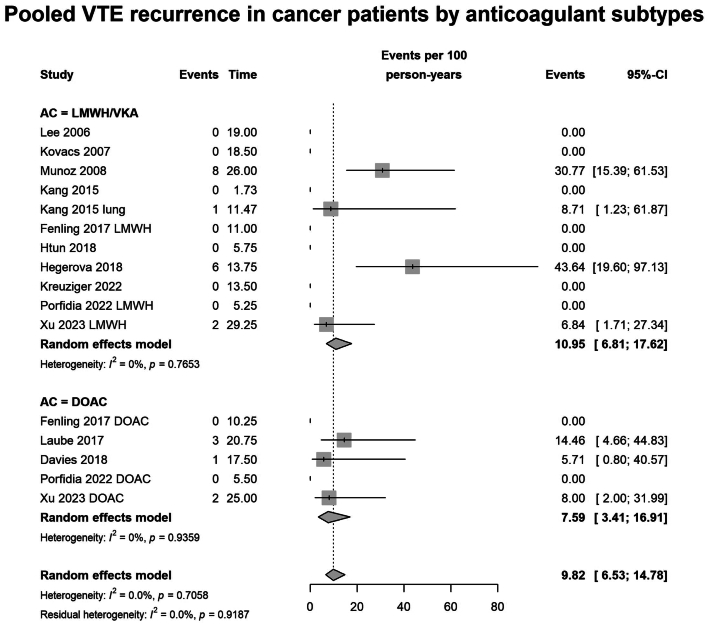
Forest plot of pooled recurrent venous thromboembolism (*VTE*) rate in patients with cancer and catheter-related thrombosis (CRT) grouped by anticoagulants, eg low-molecular-weight heparin/vitamin K antagonists (*LMWH/VKA*) vs direct oral anticoagulants (*DOACs*), expressed as events per 100 person-years.

The VTE recurrence rate was similar between LMWH/VKA compared with DOAC, with 7.59 per 100 person-years in patients receiving DOACs (95% CI, 3.41-16.91; I^2^ = 0%; n = 316 patients across 5 studies), and 10.95 per 100 person-years in those treated with LMWH/VKAs (95% CI, 6.81-17.62; I^2^ = 0%; n = 551 patients across 10 studies; *P* = .14 for subgroup difference) (Fig 4).

#### Bleeding

**Fig 5:**
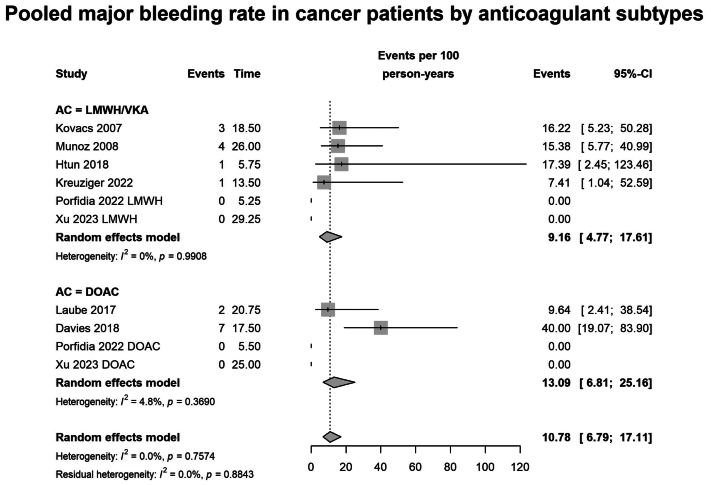
Forest plot of pooled major bleeding rate in patients with cancer and catheter-related thrombosis (CRT) grouped by anticoagulants, such as low-molecular-weight heparin/vitamin K antagonists (*LMWH/VKA*) vs direct oral anticoagulants (*DOACs*). Expressed as events per 100 person-years.

**Supplementary Fig 2:**
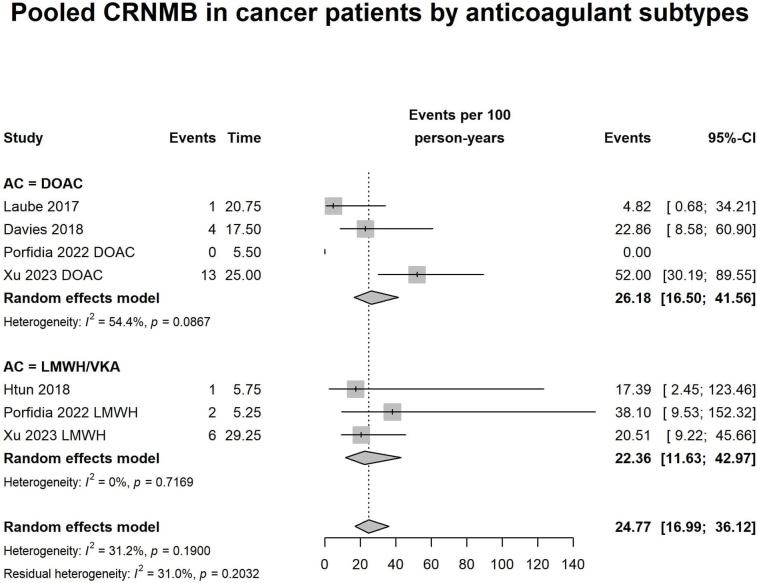
Forest plot of pooled clinically relevant nonmajor bleeding (*CRNMB*) rate in patients with cancer and catheter-related thrombosis (CRT) grouped by anticoagulants, low-molecular-weight heparin/vitamin K antagonists (*LMWH/VKA*) vs direct oral anticoagulants (*DOACs*), expressed as events per 100 person-years. *CI*, confidence interval.

The incidence rate of major bleeding was 9.16 per 100 person-years (95% CI, 6.79-17.11; I^2^ = 0%; n = 490 patients across 6 studies) in the LMWH/VKA group, and 13.09 per 100 person-years (95% CI, 6.81-25.19; I^2^ = 0%; n = 275 patients across 4 studies; *P* = .45 for subgroup difference) in the DOAC group (Fig 5). CRNMB was reported in six studies (n = 506), with no difference in pooled incidence rates between patients receiving LMWH/VKAs (22.36 per 100 person-years; 95% CI, 11.63-42.97; n = 161 patients across 3 studies) and those receiving DOACs (26.18 per 100 person-years; 95% CI, 16.50-41.56; I^2^ = 54.4%; n = 275 patients across 4 studies; *P* = .70 for subgroup difference) (Supplementary Fig 2, online only).

#### Other outcomes

Data on mortality and line removal were not pooled owing to substantial clinical and methodological heterogeneity across studies. For mortality, this related primarily to differences in follow-up duration and anticoagulation exposure, rather than outcome definition. For line removal, reporting was inconsistent, and indications for removal were variably described or absent.

#### Prolonged anticoagulation for CRT

Four studies evaluated the impact of anticoagulation duration >3 months in patients with CRT, primarily in patients with cancer.^29^^,^^48^^,^^49^^,^^51^ All studies found no decrease in recurrence with extending anticoagulation beyond 3 months compared with anticoagulation up to 3 months, particularly in patients whose catheter was removed, and the bleeding risks remained similar^29^^,^^48^^,^^49^^,^^51^ (Table).

**Table:** Results survey among intensive care units (*ICUs*) and hematology departments in the Netherlands

	Total	ICUs	Hematology departments
Total No. of hospitals included	82 (100%)	57 (100)	25 (100)
Protocol for treatment CRT present	16 (20)	5 (9)	11 (44)
Options of treatment symptomatic CRT			
LMWH + OAC upon discharge	8 (10)	2 (4)	6 (24)
DOAC	3 (4)	1 (2)	2 (8)
LWMH	41 (50)	34 (60)	7 (28)
Unfractionated heparin	3 (4)	3 (5)	0 (0)
Systemic thrombolytic	2 (2)	1 (2)	1 (4)
At the discretion of treating physician^a^	25 (31)	16 (28)	9 (36)
Treat asymptomatic CRT?			
Yes	57 (70)	42 (74)	15 (63)
No	9 (11)	7 (12)	2 (8)
At the discretion of treating physician	14 (17)	8 (14)	6 (24)
Undefined	2 (2)	0 (0)	2 (8)
Treatment of asymptomatic CRT			
DOAC	3 (4)	1 (2)	2 (8)
LMWH	32 (39)	30 (53)	2 (8)
LMWH + OAC^b^	1 (4)	0 (0)	1 (4)
Unfractionated heparin	2 (27)	2 (4)	0 (0)
Line removal alone	4 (5)	4 (7)	0 (0)
At the discretion of treating physician^c^	31 (87)	13 (23)	18 (64)
No treatment	9 (11)	7 (12)	2 (8)
Duration of treatment			
Based on clinical picture	8 (10)	7 (12)	1 (4)
≤3 months	53 (65)	30 (53)	23 (92)
>3 months	8 (10)	7 (12)	1 (4)
Undefined	13 (16)	13 (23)	0 (0)
Routine follow-up ultrasound performed	27 (33)	13 (23)	14 (56)

*CRT,* Catheter-related thrombosis; *DOAC,* direct oral anticoagulant; *LMWH,* low-molecular-weight heparin; *OAC,* oral anticoagulant; *VKA,* vitamin K antagonist.

Values are number (%).

a Either DOAC or VKA are possible.

b DOAC, LMWH, unfractionated heparin or thrombolysis are possible.

c DOAC, LMWH, unfractionated heparin, line removal alone, VKA, or no treatment are possible.

#### Fibrinolytics

Fibrinolytics alongside or sequentially with anticoagulants were used in six studies. One study reported discontinuation of fibrinolysis owing to major bleeding in one case.^20^ Reporting of key outcomes such as PE, mortality, and major bleeding was inconsistent, limiting direct comparisons. None of the studies clarified the selection criteria for fibrinolytic therapy over anticoagulation alone (Supplementary Table IV, online only).

#### Catheter removal with or without anticoagulation

Line removal, with or without subsequent anticoagulation, was reported as an intervention in four studies.^41^^,^^43^^,^^44^ Studies comparing line removal with and without anticoagulation found that adding anticoagulants decreased VTE recurrence but increased bleeding risk.^41^^,^^44^ Another study showed that not using anticoagulants led to worse outcomes,^43^ and removing the catheter before starting anticoagulants increased the risk of VTE recurrence^45^ (Supplementary Table I, online only).

#### Publication bias and sensitivity analysis

Funnel plots, generated for analyses with >10 studies, showed some asymmetry, with four studies identified as outliers^26^^,^^38^^,^^49^^,^^50^ (Supplementary Fig 3, online only). Excluding statistical outliers reduced VTE recurrence in AC-treated patients from 14.09 to 7.14 per 100 patient-years (z = 2.28; *P* = .02) and, in a second VTE analysis, from 10.95 to 2.60 per 100 patient-years (z = 2.30; *P* = .02). PE recurrence was unchanged (from 4.46 to 3.77 per 100 patient-years; z = 0.57; *P* = .57). Across subgroup meta-analyses, GLMM in comparison IV+CC produced broadly similar point estimates, but IV+CC often yielded wider CIs and greater heterogeneity (VTE overall I^2^ = 47.1% vs I^2^ = 61.3%). The largest model-related differences appeared in bleeding outcomes under DOACs (MB, 13.0 vs 20.5; CRNMB, 26.2 vs 27.3 per 100 patient-years) where the IV+CC CIs and I^2^ were notably larger. For device-specific outcomes, sensitivity analyses comparing the PICC-only and mixed (PICC/CVC) cohorts revealed similar VTE incidence in the PICC-only subgroup (5.64 per 100 patient-years; 95% CI, 2.35-13.54) and mixed studies (12.37 per 100 patient-years; 95% CI, 7.79-19.63; I^2^ = 0%; *P* = .12 for subgroup difference).

For CRNMB, PICC-only studies showed a higher pooled incidence (35.02 per 100 patient-years; 95% CI, 22.34-54.91, I^2^ = 71.8%) compared with mixed studies (14.61 per 100 patient-years; 95% CI, 7.31-29.22, I^2^ = 0%; *P* = .04).

### Survey

A total of 66 Dutch ICUs and 25 hematology departments were invited to participate in the survey. Of the 66 ICUs, 57 (86%) responded, as did all 25 (100%) hematology departments. Results are presented in Table. Protocols for CRT treatment were more commonly available in hematology departments than in ICUs (44% vs 9%; *P* = .02). In both settings, a minority CRT treatment was left to the discretion of the treating physician.

Most ICUs and hematology departments treated symptomatic CRT with LMWH, both during hospitalization and after discharge, often transitioning to oral anticoagulants. No single LMWH type was used consistently. Asymptomatic CRT was also generally treated, although hematology departments more frequently left management decisions to physician discretion (64% vs 23%; *P* < .0001). Treatment duration varied from 14 days to 6 months. Approximately one in four ICUs reported having no fixed treatment period or criteria for determining duration. Others based it on the patient's clinical course or follow-up imaging results.

Follow-up ultrasound examination during CRT treatment was more routinely performed in hematology departments than ICUs (56% vs 23%; *P* < .0001), with timing guided by CVC insertion or replacement, ICU discharge, or discontinuation of anticoagulation.

## Discussion

Findings from this systematic review on treatment of catheter related thrombosis were that, (1) among patients with CRT, LMWH/VKA and DOACs achieve comparable low VTE recurrence rates, with higher recurrence in cancer than patients without cancer; (2) major and CRNMB rates were considerable and occurred at similar rates with both treatment classes; and (3) overall, methodological quality of the included studies was low, with substantial variability across the included studies in terms of treatment type, duration, and outcome definitions.

The major findings of the survey in Dutch ICU and hematology departments are that (1) most physicians treat both symptomatic and asymptomatic CRT; (2) LMWH is mostly used as treatment for symptomatic CRT, although DOACs are also used, despite guidelines favoring LMWH—ICU patients were frequently treated with unfractionated heparin because of increased bleeding risk; and (3) there is a large variation in treatment duration, from 2 weeks ≤6 months, with the majority treating for ≤3 months.

Although previous systematic reviews have addressed related populations, there are key differences in scope and methodology compared with the present review. Two earlier reviews focused on upper extremity DVT in general, without restricting inclusion to catheter-related cases.^53^^,^^54^ Another review included only patients with cancer and CRT, but highlighted substantial heterogeneity in anticoagulation duration, follow-up periods, and outcome definitions, which prevented meta-analysis and limited the ability to draw conclusions about treatment efficacy and safety.^55^ More recent reviews focused specifically on CRT but only reported 3-month outcomes, did not assess long-term outcomes beyond 3 months, and did not evaluate alternative strategies such as fibrinolytics or catheter removal.^54^^,^^56^^,^^57^ Similarly, another systematic review on UEDVT treatment broadly but did not separately analyze CRT or assess long-term outcomes.^53^

The present review builds on these efforts by focusing specifically on CRT, incorporating more recent studies with longer follow-up and quantifying both recurrence and bleeding rates stratified by anticoagulant type and cancer status. Although no studies directly compare LMWH with DOACs for CRT, evidence from DVT populations suggests the two are generally comparable in efficacy. In acutely ill medical inpatients, randomized data informing American Society of Hematology guidance found no symptomatic VTE benefit for DOACs over LMWH and a higher risk of major bleeding, underscoring caution when extrapolating DOACs to catheter-related settings. This difference may be partly explained by our meta-analysis including predominantly hematological patients and largely based on retrospective data. Prospective CRT data are limited to a single-arm rivaroxaban study with some evidence of substantial major bleeding and a small, feasibility-limited LMWH-treated catheter-removed cohort with zero observed recurrences. Together, these studies provide limited and mixed evidence, insufficient to support DOACs over LMWH in CRT.

Two studies included in this systematic review suggest that catheter removal alone, without subsequent anticoagulation, may decrease bleeding risk, but is also associated with a greater risk of recurrent VTE. Notably, in a prospective cohort, withholding anticoagulation or providing no CRT-specific treatment was independently associated with increased mortality after adjustment for relevant covariates. However, all of the included studies were nonrandomized and mostly small, single-center cohorts, with no standardized treatment protocols. It is likely that clinicians opted to withhold anticoagulation in patients they perceived to be at high bleeding risk. This practice introduces a strong risk of confounding by indication, meaning that the apparent safety of line removal alone could reflect clinical judgment rather than a true therapeutic effect.

According to current guidelines, in select cases fibrinolytics might be considered as a treatment option, with limb-threatening ischemia or phlegmasia, provided the bleeding risk is low.^58^, ^59^, ^60^, ^61^ Based on current included articles, the role of fibrinolytics in CRT remains unclear owing to limited data, inconsistent patient selection, and variable outcomes, preventing firm conclusions about their safety or efficacy.

We performed a survey to capture the effect of the lack of clear evidence on current practice in daily care. As noted, many departments used DOACs, LMWH, or unfractionated heparin, with treatment durations ranging from 2 weeks to 6 months. Current guidelines recommend a minimum duration of 3 months or for as long as the catheter remains in place.^58^, ^59^, ^60^, ^61^ In patients with CRT, particularly those whose catheter is removed, extending anticoagulation beyond 3 months has not consistently shown a decrease in recurrence risk. Bleeding rates appear to be similar regardless of treatment duration, although data are limited and observational. These findings suggest that shorter anticoagulation courses may be sufficient in select patients, but prospective studies are needed to confirm safety.^48^^,^^49^^,^^51^^,^^52^ Evidence for asymptomatic CRT treatment is even more limited, rarely distinguishing treatment strategies or outcomes. Practice varies; some departments treating asymptomatic CRT like symptomatic cases, whereas others do not treat it at all, reflecting guideline uncertainty.

This study has several strengths. To our knowledge, it represents the most comprehensive meta-analysis on the topic. The survey offers insights into both published evidence and real-world clinical practice in the management of CRT. In addition, we evaluated treatments other than anticoagulation. Finally, a notable strength is the inclusion of both patients with cancer and patients without cancer, allowing for a comparison of treatment approaches across these distinct populations.

This review also has several limitations. The findings may be affected by confounding bias; most included observational studies lacked a control group and blinded outcome assessment. Our sensitivity analyses did demonstrate statistically significant differences after exclusion of outliers for VTE recurrence during LMWH/VKA treatment and in patients with cancer, which suggests that the overall effect estimates may be affected by outliers. In terms of device-specific outcomes, we observed similar VTE incidence in the PICC-only subgroup and mixed studies (PICC/CVC), although the pooled incidence of CRNMB was notably higher in PICC-only studies. This finding highlights the variability in bleeding outcomes depending on the device type, which may affect the interpretation of pooled estimates. Symptomatic and asymptomatic VTE cases were combined owing to the limited number of asymptomatic cases, which were generally identified incidentally through clinically indicated imaging, because systematic screening was not performed regularly. However, the impact of this limitation is likely small, given the relatively few asymptomatic cases. Most studies had a serious risk of bias and were highly variable in design, cancer type, treatment, and outcome definitions. Low event numbers, inconsistent bleeding reporting, and wide CIs further limit the reliability of the pooled estimates. In addition, data on whether events occurred during or after anticoagulation treatment were not reported consistently, precluding on-treatment analysis. The calculation of incidence rates per 100 person-years further assumes a constant event rate over time, although front-loaded recurrence patterns are common in CRT and VTE.^1^^,^^4^ This may lead to underestimation of event rates in studies with longer follow-up, even if the recurrence pattern is similar. Furthermore, the lack of data on inpatient vs outpatient status limits our ability to assess disease severity and its potential impact on VTE risk. Additionally, the studies did not consistently report the distinction between same-site and contralateral DVT, preventing a detailed analysis of whether VTE occurred in the same vessel or in a new one.

These limitations underscore the need for cautious interpretation and further investigation to confirm the robustness of these findings.

Regarding the survey, its generalizability is limited; participating hospitals were restricted to a single country. However, a range of general and academic hospitals were included. Indeed, the variability in management reported aligns with findings from a previous survey conducted among hematology centers in the Netherlands and Belgium.^62^

In summary, the current literature is limited by observational designs with small sample sizes and variation in treatment type and duration. This results in considerable variation in clinical practice.

This study highlights the need for large, prospective, well-designed randomized controlled trials focusing on specific subgroups, such as patients with active malignancy or those requiring line maintenance, to establish evidence-based treatment guidelines for CRT.

## Conclusions

Both LMWH/VKA and DOACs achieve comparable low VTE recurrence rates, with higher recurrence rates in patients with cancer than patients without cancer. Bleeding rates were considerable, but occurred at similar rates with both treatment classes. Overall, the strength of evidence is limited by observational nature and inherent risk of bias. Results of the survey indicate that both symptomatic and asymptomatic CRTs are most commonly treated with LMWH, but there is great variability in treatment modality and duration. Given these limitations and the variability in current treatment approaches, well-designed randomized controlled trials are urgently needed to provide robust, high-quality data specifically focused on CRT treatment, rather than relying on extrapolated data from DVT management.

## Author contributions

Conception and design: LO, NVE, JS, PT

Analysis and interpretation: LO, MM, ADM, APJ, BB, NVE, PT

Data collection: LO, JVD, FL, JR, GB, FVZ, KC, JS

Writing the article: LO, JVD

Critical revision of the article: LO, JVD, FL, JR, GB, FVZ, KC, MM, ADM, APJ, BB, NVE, JS, PT

Final approval of the article: LO, JVD, FL, JR, GB, FVZ, KC, MM, ADM, APJ, BB, NVE, JS, PT

Statistical analysis: LO, NVE, PT

Obtained funding: Not applicable

Overall responsibility: LO

LO and JVD contributed equally to this article and share co-first authorship.

## Funding

This work was supported by institutional resources from the 10.13039/100019573Amsterdam University Medical Centers (Amsterdam UMC), without dedicated project funding. The institution had no role in study design, data collection, analysis, decision to publish, or manuscript preparation.

## Disclosures

None.

## Appendix (online only)

Supplementary Fig 3 Funnel plot with outliers.^26^^,^^38^^,^^49^^,^^50^*AC*, anticoagulant; *CRT*, catheter-related thrombosis; *LMHW/VKA*, low-molecular-weight heparin/vitamin K antagonist; *PE*, pulmonary embolism; *VTE*, venous thromboembolism.
