# Supplementary figures and images for: Treatment of catheter related thrombosis: A systematic review, meta-analysis, and national survey
Source: J Vasc Surg Venous Lymphat Disord. 2025 Nov 29;14(2):102359. doi: 10.1016/j.jvsv.2025.102359 (PMC12796749; doi:10.1016/j.jvsv.2025.102359)

## Slide 1
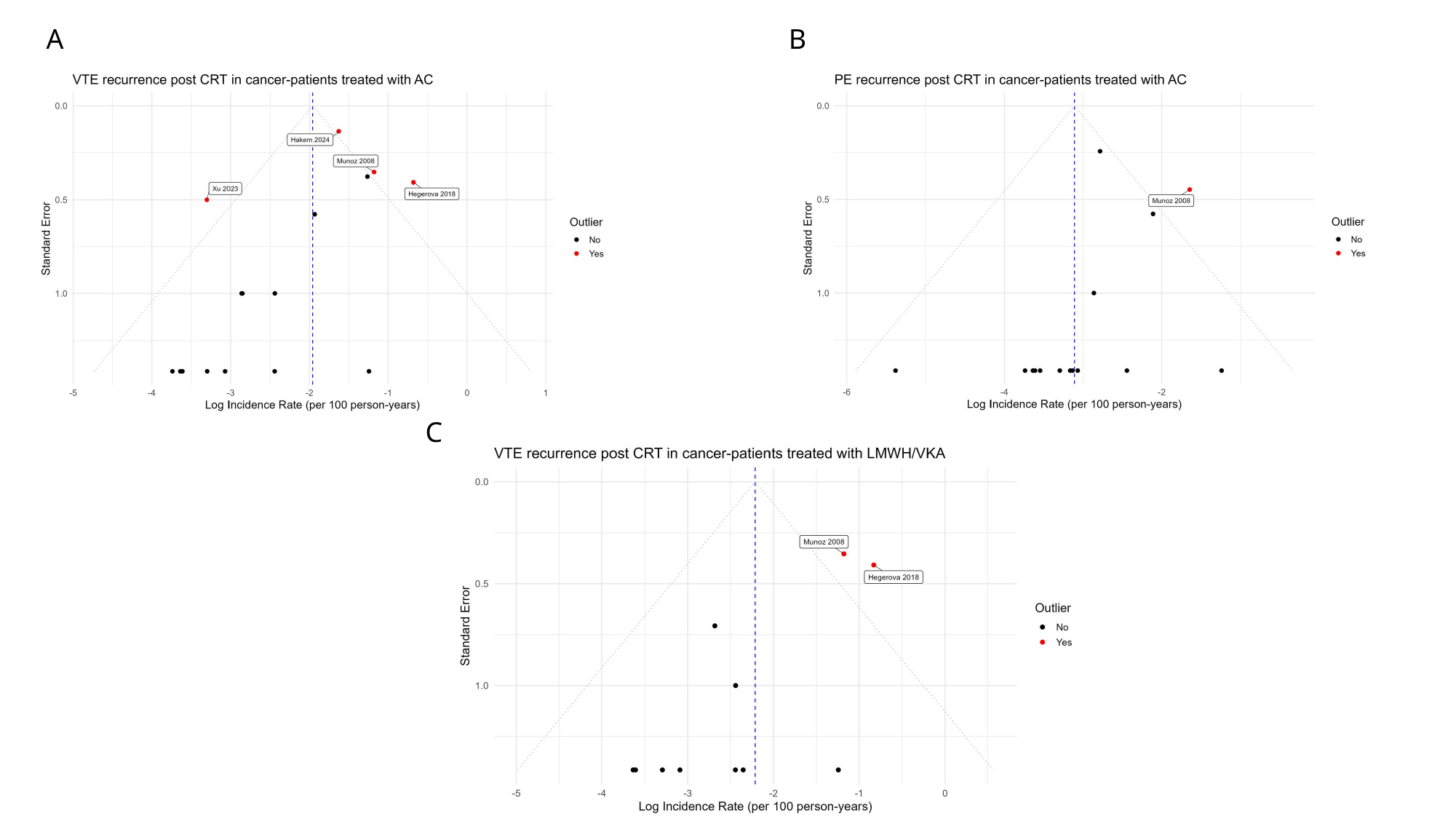

A
B
C

Supplement: Supplementary Fig 3 — Funnel plot with outliers.26,38,49,50AC, anticoagulant; CRT, catheter-related thrombosis; LMHW/VKA, low-molecular-weight heparin/vitamin K antagonist; PE, pulmonary embolism; VTE, venous thromboembolism. [file mmc1.pptx]
