# Supplementary material for: Treatment of catheter related thrombosis: A systematic review, meta-analysis, and national survey
Source: J Vasc Surg Venous Lymphat Disord. 2025 Nov 29;14(2):102359. doi: 10.1016/j.jvsv.2025.102359 (PMC12796749; doi:10.1016/j.jvsv.2025.102359)
Supplement: Supplementary Table [file mmc2.docx]

*Supplementary tables & figures*

Supplementary Table 1: PRISMA checklist

| **Section and Topic** | **Item #** | **Checklist item** | **Location where item is reported** |
| --- | --- | --- | --- |
| **TITLE** | | |  |
| Title | 1 | Identify the report as a systematic review. | Title |
| **ABSTRACT** | | |  |
| Abstract | 2 | See the PRISMA 2020 for Abstracts checklist. | Abstract |
| **INTRODUCTION** | | |  |
| Rationale | 3 | Describe the rationale for the review in the context of existing knowledge. | Introduction |
| Objectives | 4 | Provide an explicit statement of the objective(s) or question(s) the review addresses. | Introduction |
| **METHODS** | | |  |
| Eligibility criteria | 5 | Specify the inclusion and exclusion criteria for the review and how studies were grouped for the syntheses. | Methods |
| Information sources | 6 | Specify all databases, registers, websites, organisations, reference lists and other sources searched or consulted to identify studies. Specify the date when each source was last searched or consulted. | Methods |
| Search strategy | 7 | Present the full search strategies for all databases, registers and websites, including any filters and limits used. | Supplementary |
| Selection process | 8 | Specify the methods used to decide whether a study met the inclusion criteria of the review, including how many reviewers screened each record and each report retrieved, whether they worked independently, and if applicable, details of automation tools used in the process. | Methods |
| Data collection process | 9 | Specify the methods used to collect data from reports, including how many reviewers collected data from each report, whether they worked independently, any processes for obtaining or confirming data from study investigators, and if applicable, details of automation tools used in the process. | Methods – data extraction |
| Data items | 10a | List and define all outcomes for which data were sought. Specify whether all results that were compatible with each outcome domain in each study were sought (e.g. for all measures, time points, analyses), and if not, the methods used to decide which results to collect. | Methods – data extraction |
|  | 10b | List and define all other variables for which data were sought (e.g. participant and intervention characteristics, funding sources). Describe any assumptions made about any missing or unclear information. | Methods – data extraction |
| Study risk of bias assessment | 11 | Specify the methods used to assess risk of bias in the included studies, including details of the tool(s) used, how many reviewers assessed each study and whether they worked independently, and if applicable, details of automation tools used in the process. | Methods – Quality assessment |
| Effect measures | 12 | Specify for each outcome the effect measure(s) (e.g. risk ratio, mean difference) used in the synthesis or presentation of results. | Methods – statistical analysis |
| Synthesis methods | 13a | Describe the processes used to decide which studies were eligible for each synthesis (e.g. tabulating the study intervention characteristics and comparing against the planned groups for each synthesis (item #5)). | Methods – statistical analysis |
|  | 13b | Describe any methods required to prepare the data for presentation or synthesis, such as handling of missing summary statistics, or data conversions. | Methods – statistical analysis |
|  | 13c | Describe any methods used to tabulate or visually display results of individual studies and syntheses. | Methods – statistical analysis |
|  | 13d | Describe any methods used to synthesize results and provide a rationale for the choice(s). If meta-analysis was performed, describe the model(s), method(s) to identify the presence and extent of statistical heterogeneity, and software package(s) used. | Methods – statistical analysis |
|  | 13e | Describe any methods used to explore possible causes of heterogeneity among study results (e.g. subgroup analysis, meta-regression). | Methods – statistical analysis |
|  | 13f | Describe any sensitivity analyses conducted to assess robustness of the synthesized results. | Methods – statistical analysis |
| Reporting bias assessment | 14 | Describe any methods used to assess risk of bias due to missing results in a synthesis (arising from reporting biases). | Not applicable |
| Certainty assessment | 15 | Describe any methods used to assess certainty (or confidence) in the body of evidence for an outcome. | Not applicable |
| **RESULTS** | | |  |
| Study selection | 16a | Describe the results of the search and selection process, from the number of records identified in the search to the number of studies included in the review, ideally using a flow diagram. | Results-systematic review/supplementary |
|  | 16b | Cite studies that might appear to meet the inclusion criteria, but which were excluded, and explain why they were excluded. | Figure 1 |
| Study characteristics | 17 | Cite each included study and present its characteristics. | Table 1, Table 2 |
| Risk of bias in studies | 18 | Present assessments of risk of bias for each included study. | Supplementary table 2/Figure 5 |
| Results of individual studies | 19 | For all outcomes, present, for each study: (a) summary statistics for each group (where appropriate) and (b) an effect estimate and its precision (e.g. confidence/credible interval), ideally using structured tables or plots. | Figure 2-3, Supplementary Figures 2-3 |
| Results of syntheses | 20a | For each synthesis, briefly summarise the characteristics and risk of bias among contributing studies. | Figure 5 |
|  | 20b | Present results of all statistical syntheses conducted. If meta-analysis was done, present for each the summary estimate and its precision (e.g. confidence/credible interval) and measures of statistical heterogeneity. If comparing groups, describe the direction of the effect. | Results – recurrence & bleeding |
|  | 20c | Present results of all investigations of possible causes of heterogeneity among study results. | Results – recurrence & bleeding |
|  | 20d | Present results of all sensitivity analyses conducted to assess the robustness of the synthesized results. | Results – quality assessment & sensitivity analysis |
| Reporting biases | 21 | Present assessments of risk of bias due to missing results (arising from reporting biases) for each synthesis assessed. | Figure 5, Table 1 |
| Certainty of evidence | 22 | Present assessments of certainty (or confidence) in the body of evidence for each outcome assessed. | Figure 5, Table 1 |
| **DISCUSSION** | | |  |
| Discussion | 23a | Provide a general interpretation of the results in the context of other evidence. | Discussion |
|  | 23b | Discuss any limitations of the evidence included in the review. | Discussion |
|  | 23c | Discuss any limitations of the review processes used. | Discussion |
|  | 23d | Discuss implications of the results for practice, policy, and future research. | Discussion |
| **OTHER INFORMATION** | | |  |
| Registration and protocol | 24a | Provide registration information for the review, including register name and registration number, or state that the review was not registered. | Methods - search |
|  | 24b | Indicate where the review protocol can be accessed, or state that a protocol was not prepared. | Methods - search |
|  | 24c | Describe and explain any amendments to information provided at registration or in the protocol. | Not applicable |
| Support | 25 | Describe sources of financial or non-financial support for the review, and the role of the funders or sponsors in the review. | Not applicable |
| Competing interests | 26 | Declare any competing interests of review authors. | Conflict of interest statement |
| Availability of data, code and other materials | 27 | Report which of the following are publicly available and where they can be found: template data collection forms; data extracted from included studies; data used for all analyses; analytic code; any other materials used in the review. | Supplementary |

Supplementary Table 2: Search syntaxes

PubMed (2271)

| Search | Query | Results |
| --- | --- | --- |
| #7 | #6 NOT (("Case Reports"[Publication Type] OR "case report*"[tiab] OR "case stud*"[tiab] OR "case histor*"[tiab] OR "case serie*"[tiab]) OR ("Congress" [Publication Type] OR "Letter" [Publication Type] OR "Lecture Note" [Publication Type])) | [2,271](https://pubmed.ncbi.nlm.nih.gov/?term=%236+NOT+%28%28%22Case+Reports%22%5BPublication+Type%5D+OR+%E2%80%9Ccase+report%2A%E2%80%9D%5Btiab%5D+OR+%E2%80%9Ccase+stud%2A%E2%80%9D%5Btiab%5D+OR+%E2%80%9Ccase+histor%2A%E2%80%9D%5Btiab%5D+OR+%E2%80%9Ccase+serie%2A%E2%80%9D%5Btiab%5D%29+OR+%28%22Congress%22+%5BPublication+Type%5D+OR+%22Letter%22+%5BPublication+Type%5D+OR+%22Lecture+Note%22+%5BPublication+Type%5D%29%29&sort=relevance&ac=no&otool=inlvulib) |
| #6 | #5 NOT (("Adolescent"[Mesh] OR "Child"[Mesh] OR "Infant"[Mesh] OR "adolescen*"[tiab] OR "child*" [tiab] OR "schoolchild*"[tiab] OR "infant*"[tiab] OR "girl*"[tiab] OR "boy"[tiab] OR "boys"[tiab] OR "teen*"[tiab] OR "youth*"[tiab] OR "pediatr*"[tiab] OR "paediatr*"[tiab] OR "pube*"[tiab]) NOT ("Adult"[Mesh] OR "adult*"[tiab] OR "man"[tiab] OR "men"[tiab] OR "woman"[tiab] OR "women"[tiab])) | [2,413](https://pubmed.ncbi.nlm.nih.gov/?term=%235+NOT+%28%28%22Adolescent%22%5BMesh%5D+OR+%22Child%22%5BMesh%5D+OR+%22Infant%22%5BMesh%5D+OR+%E2%80%9Cadolescen%2A%E2%80%9D%5Btiab%5D+OR+%E2%80%9Cchild%2A%E2%80%9D+%5Btiab%5D+OR+%E2%80%9Cschoolchild%2A%E2%80%9D%5Btiab%5D+OR+%E2%80%9Cinfant%2A%E2%80%9D%5Btiab%5D+OR+%E2%80%9Cgirl%2A%E2%80%9D%5Btiab%5D+OR+%E2%80%9Cboy%E2%80%9D%5Btiab%5D+OR+%E2%80%9Cboys%E2%80%9D%5Btiab%5D+OR+%E2%80%9Cteen%2A%E2%80%9D%5Btiab%5D+OR+%E2%80%9Cyouth%2A%E2%80%9D%5Btiab%5D+OR+%E2%80%9Cpediatr%2A%E2%80%9D%5Btiab%5D+OR+%E2%80%9Cpaediatr%2A%E2%80%9D%5Btiab%5D+OR+%E2%80%9Cpube%2A%E2%80%9D%5Btiab%5D%29+NOT+%28%22Adult%22%5BMesh%5D+OR+%E2%80%9Cadult%2A%E2%80%9D%5Btiab%5D+OR+%E2%80%9Cman%E2%80%9D%5Btiab%5D+OR+%E2%80%9Cmen%E2%80%9D%5Btiab%5D+OR+%E2%80%9Cwoman%E2%80%9D%5Btiab%5D+OR+%E2%80%9Cwomen%E2%80%9D%5Btiab%5D%29%29&sort=relevance&ac=no&otool=inlvulib) |
| #5 | #1 AND #2 AND #3 AND #4 | [2,767](https://pubmed.ncbi.nlm.nih.gov/?term=%231+AND+%232+AND+%233+AND+%234&sort=relevance&ac=no) |
| #4 | Treat*[tiab] OR "central line removal"[tiab] OR "standard of care"[tiab] OR "surgery"[Subheading] OR "Surgical Procedures, Operative"[Mesh] OR "Surgeons"[Mesh] OR "Perioperative Period"[Mesh] OR "Perioperative Care"[Mesh] OR surg*[tiab] OR operat*[tiab] OR perioperati*[tiab] OR incisi*[tiab] OR extracti*[tiab] OR excisi*[tiab] OR resect*[tiab] OR invasive*[tiab] OR restorati*[tiab] OR intraoperative[tiab] OR ( ("Heparin, Low-Molecular-Weight"[Mesh] OR "Heparin"[Mesh] OR "heparin"[tiab] OR "nadroparin"[tiab] OR "LMWH"[tiab] OR "enoxaparin"[tiab] OR "Dalteparin"[tiab]) OR ("Anticoagulants"[Mesh] OR "Anticoagulants"[Pharmacological Action] OR "anticoagula*"[tiab] OR "anti coagula*"[tiab]) OR ("Factor Xa Inhibitors"[Mesh] OR "Direct acting oral anticoagulant"[tiab] OR "factor Xa inhibitor*"[tiab] OR "DOAC"[tiab] OR "Dabigatran"[tiab] OR "Rivaroxaban"[tiab] OR "Apixaban"[tiab] OR "Edoxaban"[tiab]) OR ("Coumarins"[Mesh] OR "Coumarin*"[tiab] OR "Vitamin K antagonist"[tiab] OR "warfarin"[tiab] OR "acenocoumarol"[tiab] OR "phenproucomon"[tiab]) OR "antithromb*"[tiab] OR "anti thromb*"[tiab] OR "DOAC*"[tiab] OR "warfarin*"[tiab] OR "acenocoumarol"[tiab] OR "Sintrom"[tiab] OR "prasugrel"[tiab] OR "Efient"[tiab] OR "rivaroxaban"[tiab] OR "Xarelto"[tiab] OR "edoxaban"[tiab] OR "Lixiana"[tiab] OR "coumarin*"[tiab] OR "cumarin*"[tiab] OR "apixaban"[tiab] OR "eliquis"[tiab] OR "dabigatran"[tiab] OR "Pradaxa"[tiab] OR "Fenprocoumon"[tiab] OR "Marcoumar"[tiab] OR "Nadroparin"[tiab] OR "Fraxiparine"[tiab] OR "Heparin"[tiab] OR "Long acting molecular weight heparin"[tiab] OR "Tinzaparine"[tiab] OR "Innohep"[tiab] OR "Enoxaparine"[tiab] OR "Clexane"[tiab] OR "Dalteparine"[tiab] OR "Fragmin"[tiab] OR "Plasminogen Activators"[Mesh] OR "Thrombectomy"[Mesh] OR "Thrombin/surgery"[Mesh] OR "thrombolysis"[tiab] OR "Alteplase"[tiab] OR "thrombolytic"[tiab] OR "plasminogen activator*"[tiab] OR "Thrombectom*"[tiab] OR "Eminase"[tiab] OR "retavase"[tiab] OR "streptase"[tiab] OR "tPA"[tiab] OR "Anistreplase"[tiab] OR "reteplase"[tiab] OR "streptokinase"[tiab] OR "kabikinase"[tiab] OR "Urokinase"[tiab] ) | [12,295,736](https://pubmed.ncbi.nlm.nih.gov/?term=longquery5ba98dea6797792a619f&sort=relevance&ac=no) |
| #3 | "compar*"[tiab] OR "versus"[tiab] OR "contrast"[tiab] OR "vs"[tiab] | [8,673,240](https://pubmed.ncbi.nlm.nih.gov/?term=%E2%80%9Ccompar%2A%E2%80%9D%5Btiab%5D+OR%0A%E2%80%9Cversus%E2%80%9D%5Btiab%5D+OR%0A%22contrast%22%5Btiab%5D+OR%0A%E2%80%9Cvs%E2%80%9D%5Btiab%5D&sort=relevance&ac=no) |
| #2 | "Embolism and Thrombosis"[Mesh] OR "throm*"[tiab] OR "embolism*"[tiab] OR "CRT"[tiab] | [665,218](https://pubmed.ncbi.nlm.nih.gov/?term=%22Embolism+and+Thrombosis%22%5BMesh%5D+OR%0A%E2%80%9Cthrom%2A%E2%80%9D%5Btiab%5D+OR%0A%E2%80%9Cembolism%2A%E2%80%9D%5Btiab%5D+OR%0A%22CRT%22%5Btiab%5D&sort=relevance&ac=no) |
| #1 | "Catheterization, Central Venous/adverse effects"[Mesh] OR "Central Venous Catheters"[Mesh] OR "Vascular Access Devices"[Mesh] OR (central[tiab] AND catheter*[tiab]) OR "subclavian vein catheter*"[tiab] OR "jugular vein catheter*"[tiab] OR "femoral vein catheter*"[tiab] OR "cvc"[tiab] OR "central venous access*"[tiab] OR "central line*"[tiab] OR "central access device*"[tiab] OR "vascular access*"[tiab] OR PICC[tiab] | [52,475](https://pubmed.ncbi.nlm.nih.gov/?term=%E2%80%9CCatheterization%2C+Central+Venous%2Fadverse+effects%22%5BMesh%5D+OR%0A%22Central+Venous+Catheters%22%5BMesh%5D+OR%0A%22Vascular+Access+Devices%22%5BMesh%5D+OR%0A%28central%5Btiab%5D+AND+catheter%2A%5Btiab%5D%29+OR%0A%E2%80%9Csubclavian+vein+catheter%2A%E2%80%9D%5Btiab%5D+OR%0A%E2%80%9Cjugular+vein+catheter%2A%E2%80%9D%5Btiab%5D+OR%0A%E2%80%9Cfemoral+vein+catheter%2A%E2%80%9D%5Btiab%5D+OR%0A%22cvc%22%5Btiab%5D+OR+%0A%22central+venous+access%2A%22%5Btiab%5D+OR+%0A%E2%80%9Ccentral+line%2A%E2%80%9D%5Btiab%5D+OR%0A%E2%80%9Ccentral+access+device%2A%E2%80%9D%5Btiab%5D+OR%0A%22vascular+access%2A%22%5Btiab%5D+OR%0APICC%5Btiab%5D&sort=relevance&ac=no) |

Embase (3010)

| Search | Query | Results |
| --- | --- | --- |
| #7 | #6 NOT ('case report'/exp OR (“case report*” OR “case stud*” OR “case histor*” OR “case serie*”):ti,ab,kw OR 'conference abstract'/it OR 'conference review'/it OR 'conference paper'/it OR 'editorial'/it OR 'erratum'/it OR 'letter'/it OR 'note'/it OR 'short survey'/it OR 'tombstone'/it OR 'chapter'/it) | 3010 |
| #6 | #5 NOT (('juvenile'/exp OR 'embryo'/exp OR 'fetus'/exp OR (‘adolescen*’ OR ‘child*’ OR ‘schoolchild*’ OR ‘infant*’ OR ‘girl*’ OR ‘boy’ OR ‘boys’ OR ‘teen*’ OR ‘youth*’ OR ‘newborn’ OR ‘pediatr*’ OR ‘paediatr*’ OR ‘pube*’):ti,ab,kw) NOT ('adult'/exp OR (‘adult*’ OR ‘man’ OR ‘men’ OR ‘woman’ OR ‘women’):ti,ab,kw)) | 5419 |
| #5 | #1 AND #2 AND #3 AND #4 | 6121 |
| #4 | (Treat* OR "central line removal" OR "standard of care"):ti,ab,kw OR ('surgery'/exp OR 'surgeon'/exp OR (surg* OR operat* OR perioperati* OR incisi* OR extracti* OR excisi* OR resect* OR invasive* OR restorati* OR intraoperative):ti,ab,kw) OR 'low molecular weight heparin'/exp OR 'heparin'/exp OR ("heparin" OR "nadroparin" OR "LMWH" OR "enoxaparin" OR "Dalteparin"):ti,ab,kw OR 'anticoagulant agent'/exp OR ("anticoagula*" OR "anti coagula*"):ti,ab,kw OR ("Direct acting oral anticoagulant" OR "factor Xa inhibitor*" OR "DOAC" OR "Dabigatran" OR "Rivaroxaban" OR "Apixaban" OR "Edoxaban"):ti,ab,kw OR 'coumarin derivative'/exp OR ("Coumarin*" OR "Vitamin K antagonist" OR "warfarin" OR "acenocoumarol" OR "phenproucomon" OR "antithromb*" OR "anti thromb*" OR "DOAC*" OR "warfarin*" OR "acenocoumarol" OR "Sintrom" OR "prasugrel" OR "Efient" OR "rivaroxaban" OR "Xarelto" OR "edoxaban" OR "Lixiana" OR "coumarin*" OR "cumarin*" OR "apixaban" OR "eliquis" OR "dabigatran" OR "Pradaxa" OR "Fenprocoumon" OR "Marcoumar" OR "Nadroparin" OR "Fraxiparine" OR "Heparin" OR "Long acting molecular weight heparin" OR "Tinzaparine" OR "Innohep" OR "Enoxaparine" OR "Clexane" OR "Dalteparine" OR "Fragmin"):ti,ab,kw OR 'plasminogen activator'/exp OR 'thrombectomy'/exp OR ("thrombolysis" OR "Alteplase" OR "thrombolytic" OR "plasminogen activator*" OR "Thrombectom*" OR "Eminase" OR "retavase" OR "streptase" OR "tPA" OR "Anistreplase" OR "reteplase" OR "streptokinase" OR "kabikinase" OR "Urokinase"):ti,ab,kw | 16,626,607 |
| #3 | ("compar*" OR "versus" OR "contrast" OR "vs"):ti,ab,kw | 11,691,051 |
| #2 | 'thromboembolism'/exp OR ("throm*" OR "embolism*" OR "CRT"):ti,ab,kw | 1,153,533 |
| #1 | 'central venous catheterization'/exp OR 'central venous catheter'/exp OR 'vascular access device'/exp OR ((central AND catheter*) OR "subclavian vein catheter*" OR "jugular vein catheter*" OR "femoral vein catheter*" OR "cvc" OR "central venous access*" OR "central line*" OR "central access device*" OR "vascular access*" OR PICC):ti,ab,kw | 94,437 |

Cochrane (936)

| Search | Query | Results |
| --- | --- | --- |
| #6 | #5 NOT ((adolescen* OR child* OR schoolchild* OR infant* OR girl* OR boy OR boys OR teen* OR youth* OR pediatr* OR paediatr* OR pube*):ti,ab,kw NOT (adult* OR man OR men OR woman OR women):ti,ab,kw) | 936 |
| #5 | #1 AND #2 AND #3 AND #4 | 1041 |
| #4 | (Treat* OR central NEXT line NEXT removal OR standard NEXT of NEXT care OR surg* OR operat* OR perioperati* OR incisi* OR extracti* OR excisi* OR resect* OR invasive* OR restorati* OR intraoperative):ti,ab,kw OR (heparin OR nadroparin OR LMWH OR enoxaparin OR Dalteparin):ti,ab,kw OR (anticoagula* OR anti NEXT coagula*):ti,ab,kw OR (Direct NEXT acting NEXT oral NEXT anticoagulant OR factor NEXT Xa NEXT inhibitor* OR DOAC OR Dabigatran OR Rivaroxaban OR Apixaban OR Edoxaban):ti,ab,kw OR (Coumarin* OR Vitamin NEXT K NEXT antagonist OR warfarin OR cenocoumarol OR phenproucomon):ti,ab,kw OR (antithromb* OR anti NEXT thromb* OR DOAC* OR warfarin* OR acenocoumarol OR Sintrom OR prasugrel OR Efient OR rivaroxaban OR Xarelto OR edoxaban OR Lixiana OR coumarin* OR cumarin* OR apixaban OR eliquis OR dabigatran OR Pradaxa OR Fenprocoumon OR Marcoumar OR Nadroparin OR Fraxiparine OR Heparin OR Long NEXT acting NEXT molecular NEXT weight NEXT heparin OR Tinzaparine OR Innohep OR Enoxaparine OR Clexane OR Dalteparine OR Fragmin OR thrombolysis OR Alteplase OR thrombolytic OR plasminogen NEXT activator* OR Thrombectom* OR Eminase OR retavase OR streptase OR tPA OR Anistreplase OR reteplase OR streptokinase OR kabikinase OR Urokinase):ti,ab,kw | 1353828 |
| #3 | (compar* OR versus OR contrast OR vs):ti,ab,kw | 1210732 |
| #2 | (throm* OR embolism* OR CRT):ti,ab,kw | 76531 |
| #1 | (central AND catheter*):ti,ab,kw OR (subclavian NEXT vein NEXT catheter* OR jugular NEXT vein NEXT catheter* OR femoral NEXT vein NEXT catheter* OR cvc OR central NEXT venous NEXT access* OR central NEXT line* OR central NEXT access NEXT device* OR vascular NEXT access* OR PICC):ti,ab,kw | 8013 |

Supplementary Table 3. Outcomes of studies included in the systematic review

| Study | CRT (n) | VTE recurrence (n) | PE post CRT (%) | Bleeding criteria | MB (n) | CRNMB (n) | Mortality (%) |
| --- | --- | --- | --- | --- | --- | --- | --- |
| Chang (1996) | 11 | - | - | - | - | - | - |
| Aburahma (1996) | 6 | - | - | - | 0 | - | - |
| Schindler (1999) | 18 | 4 | - | ISTH-like | 1 | 4 | 0% |
| Frank  (2000) | 112 | 0 | 0 | - | - | - | 4,9% |
| Schimp (2003) | 13 | 2 | 0 | - | - | - | 0% |
| Lee (2006) | 19 | 0 | 0 | - | - | - | 47.4% |
| Kovacs  (2007) | 74 | 0 | 0 | ISTH | 4 | - | 9,5%;1,4% due to MB |
| Frizelli (2008) | 386 | 1 | 0.3% | - | - | - | - |
| Munoz (2008) | 228 | 7.7% | CRT, cancer-related: 3.8%  CRT, non-cancer related: 1.6% | ISTH-like | 3.8% (including 1 fatal)  CRT, non-cancer 3.1% | - | Cancer: 16%;  Non-cancer CRT 4.8% |
| Tran (2010) | 39 | - | - | - | 0 | - | 47% |
| Chang (2011) | 31 | 0 | - | - | 0 | - | 0% |
| Delluc  (2015) | 99 | 7.1% | 3% | ISTH | 2 on AC 3.7 per 100-pt years (95% CI −0.1-9.0) | 1 on AC | - |
| Oliver  (2015) | 21 | - | - | CTCAE | All bleeding AC 24%, 14,3% grade 3,  No AC  All bleeding: 7% grade 2 | | Overall: AC 33%, no AC 71% |
| Kang (2015) | 8 | 0 | 0 | - | - | - | - |
| Kang (2015) (lung) | 17 | 13% | 0 | - | - | - | - |
| Guillet  (2016) | 16 | 0 | - | - | - | - | 0 |
| Fenling  (2017) | 84 | 0 | 0 | - | All bleeding 3 mo: DOAC: 7.3%, VKA/LMWH: 11.4%) | | 0 |
| Laube  (2017) | 83 | 3.6% | - | - | 2.4% | 1.2% | 7.2% |
| Underhill (2017) | 161 | - | Therapeutic AC: 8.1% | ISTH-like | Therapeutic AC:  All bleeding 29.5%;  Major: 16.4% | | Therapeutic AC: 23% |
| Htun  (2018) | 23 | 0 | 0 | ISTH | 4% | 4% | 9% |
| Hegerova (2018) | 50 | 12% | 10%(asymptomatic PE) | - | - | 4% | 0 |
| Davies  (2018) | 70 | 1.4% | 1.4% | ISTH | 10% | 5.7% | 1.4% |
| Mansour (2018) | 47 | 0% | 9.2% | - | - | - | - |
| Liu (2018) | 85 | Incl AC: 0%  Excl AC: 4.8% | Incl AC: 1.6% Excl AC: 0% | - | Incl AC: 28.5%  Excl AC: 4.8% | - | 0% |
| Shatzel (2018) | 83 | Incl AC : 0%  Excl AC 6.3% | Incl AC: 0%  Excl AC 4.5% | - | Incl AC: 28.6%  Excl AC: 4.7% | - | 0% |
| Scamuffa (2020) | 50 | 4% | - | - | - | 4% | - |
| Kreuziger (2021) | 663 | 15% | 2.6% | ISTH | Overall bleeding (minor + major combined):  AC + line removal: 6.3%  AC only: 2.1 %  Line removal only: 0.9% | | 34% |
| Kreuziger (2022) | 27 | 0% | - | ISTH | 5% | - | 0 |
| Porfidia  (2022) | 74 | 0% in all 3 groups | 0% | ISTH | 0% in all 3 groups | DOAC: 0%  LMWH: 9.5%  Fonda 3.2% | 0 in all 3 groups |
| Turrian (2022) | 60 | d/c AC 5%  Cont. AC 6.3% | - | ISTH | d/c AC 0 %  Cont. AC 6.3% | - | 1y: d/c AC 11.6%  Cont AC 19.9  2y: d/c AC 56.3%  d/c AC 78.%1 |
| Lipe (2022) | 257 | 3 mo: 7% 1 y: 10.1% | 0.4% | - | - | - | 3 mo: 13.2%  1 y: 30.7% |
| Hakem (2023) | 200 | Overall: 25.5% | - | ISTH | 4 (2%) | 17 (8.5%) | Overall: 38% |
| Xu (2023) | 127 | 2% in both groups (4% in total) | 8.5% | ISTH | 0 | 8.8% | 1.4% |
| Ngo  (2024) | 198 | PE/DVT combined:  AC: 14%  Non-AC:16% | | - | AC: 9%  No-AC: 8% | - | AC: 2%  No-AC: 0% |

Supplementary Table 3. Study outcomes. AC= anticoagulation, LMWH = low molecular weight heparin, UFH= unfractionated heparin, VKA = vitamin K antagonist, DOAC =direct oral anticoagulant, ISTH= International Society on Thrombosis and Haemostasis. PE = Pulmonary Embolism. CTCAE = Common Terminology Criteria for Adverse Events (CTCAE) Missing data is indicated with ‘-’.

Questionnaire used

1. What treatment is given in a case of symptomatic catheter-related thrombosis? (drug, duration)
2. What treatment is given in a case of asymptomatic catheter-related thrombosis? (drug, duration)
3. What criteria are used to determine the duration of treatment for catheter-related thrombosis?
4. Is routine follow-up ultrasound performed during treatment

Supplementary Table 4: Study design and patient characteristics

| Author (year) (reference) | Study Design | Patient population | Patients  (n) | CRT (n) and type (symp/asymp) | Type of AC | Follow-up median/mean (months) |
| --- | --- | --- | --- | --- | --- | --- |
| Chang et al (1996) | Prospective single center | Hemat (54%),  Solid malign (31%),  Benign (8%) | 11 | 11,  Symptomatic | Fibrinolytics -> UFH -> VKA (100%) | - |
| Aburahma et al.  (1996) | Retrospective single center | Nutritional support(50%), Cardiac (50%) | 6 | 6  Symptomatic | Line removal+ Hep+VKA (3, 50%)  Urokinase +Hep+VKA 3 (50%) | 36 (14-53) |
| Schindler et al.  (1999) | Retrospective single center | Solid malign (78%); Hemat (22%) | 51 | 51  Symptomatic | Urokinase+Hep+VKA (100%) | - |
| Frank et al.  (2000) | Retrospective Single center | Hemat (26%), solid malign (70%)  Other (4%) | 112 | 112, - | VKA, Hep, Hep+VKA, AC not specified | 6 or until death |
| Schimp (2003) | Retrospective Single center | Solid malign (gyn) (100%) | 13 | 13,  Symptomatic | Urokinase (8%),  UFH → VKA (62%)  LMWH (85%)  UFH (8%) | - |
| Lee et al (2006) | Prospective Single center | Hemat (34%), Solid malign (66%) | 19 | 19  Symptomatic | VKA (42%), LMWH (42%), UFH (5%) | 13 |
| Kovacs et al.   (2007) | Prospective, multicenter | Solid malign  (100%) | 74 | 74  Symptomatic | LMWH+VKA (100%) | 3 |
| Frizelli (2008) | Prospective, multicenter | Cardiac (100%); | 386 | 386,  - | Hep +VKA (100%) | 3 |
| Munoz, 2008 | Prospective  Multicenter | Catheter + non catheter UEDVT combined: Solid malign (100%), of which  Lung (22%), GI (33%), breast (13%) GI | 104 | 104,  Symptomatic | LMWH (77%), VKA (23%) | At least 3 |
| Munoz, 2008 | Prospective  Multicenter | Benign (100%) | 124 | 124,  Symptomatic | LMWH (48%), VKA(52%) | At least 3 |
| Tran (2010) | Retrospective Single center | Hemat (100%) | 39 | 39,  Symptomatic | Fibrinolytics (13%) -> VKA/LMWH (100%) | Median 20 |
| Chang (2011) | Prospective single center | Hemat (58%)  Solid malign (26%)  Congenital/Immune Disorders (16%) | 33 | 31,  Symptomatic | Fibrinolytics (100%) -> VKA/LMWH (100%) | 6 |
| Delluc et al.  (2015) | Retrospective  Single center | Solid malign (92%)  Hemat (8%) | 94 | 94  Symptomatic | LMWH (95%)  VKA (4%)  DOAC (1%) | Median 21 (0-83) |
| Oliver et al.  (2015)  (1) | Retrospective  Multicenter | Hemat (100%) | 35 | 35, - | LMWH/UFH 21 (100%) | Median 6 (0-25) |
| Kang (2015) | Retrospective Single center | Solid malign (breast) (100%) | 8 | 16,  Symptomatic | LMWH (100%) | 2.25 |
| Kang (2015) | Retrospective Single center | Solid malign (lung) (100%) | 7 | 7,  Symptomatic | LMWH (100%) | 8.2 |
| Guillet et al.  (2016) | Retrospective Single center | Prolonged I.V. antibiotic therapy (100%) | 16 | 16,  Symptomatic | Line removal --> LMWH/ VKA (100%)) | 20 |
| Fenling Fan et al.  (2017) | Prospective single centre | Unspecified malignancy | 84 | 84,  Symptomatic | LMWH/VKA ( 48%), DOAC (52%) | 3 |
| Laube et al.  (2017) | Retrospective Single center | Unspecified malignancy | 83 | 83  Symptomatic: 42  Asymptomatic: 41 | DOAC (100%) | Up to 3 |
| Underhill (2017) | Retrospective Single center | Critically ill patients with CRT | 161 | 161,  symptomatic | Therapeutic AC (76%)  Prophylactic AC (14%)  No AC ( 10%) |  |
| Htun et al.  (2018) | Retrospective Single center | Hemat (100%) | 23 | 23,  Symptomatic | LMWH (78%), No AC ( 22%) | Up to 6 |
| Hegerova et al 2018 | Retrospective Single center | Hemat (100%) | 50 | 50,  Symptomatic 16; Asymptomatic 34 | LMWH (86%)  +VKA (56%)  No AC (12%) | Up to 4 |
| Shatzel et al.  (2018) | Retrospective Single center | Hemat (100%) | 83 | 83, - | Catheter removal +AC: (25%) LMWH (11%) VKA (8%) DOAC (8%)  Catheter removal alone (75%) | - |
| Davies et al.  (2018) | Prospective Multicenter | Solid malign (71%)  Other (29%) | 70 | 70,  Symptomatic | DOAC (100%) | Up to 6 |
| Mansour (2018) | Retrospective Single center | Hemat (23%)  Solid malign (77%) | 47 | 47,  Symptomatic | LMWH, warfarin | 3.25 |
| Scamuffa (2020) | Retrospective Single center | Hemat (100%) | 50 | 50,  Symptomatic | Therapeutic LMWH or fondaparinux 7,5 mg (72%)  50 % LMWH of fondaparinux 5 mg (16%); No AC (12%) | - |
| Baumann Kreuziger (2021) | Retrospective Multicenter | Hemat (100%) | 663 | 663, - | LMWH (77%), VKA (7%),  DOAC (6%) | Median 11.4 |
| Liu et al. (2021) | Retrospective Single center | Hemat (100%) | 85 | 85, - | line removal + AC, 26% (LMWH, VKA or DOAC);  Line removal (74%) | - |
| Baumann Kreuziger (2022) | Prospective Multicenter | Hemat (70%), Solid malign (30%) | 27 | 27, - | LMWH for one month + line removal (100%) | Median 6 |
| Lipe (2022) | Retrospective Single center | Hemat (54%), Solid malign (43%)  Other (5%) | 257 | 257,  Symptomatic | LMWH (49%), DOAC (9%), VKA (2%) | 55,3 |
| Porfidia et al.  (2022) | Retrospective Single center | Solid malign (gyn, breast) 89% | 74 | 74,  - | DOAC (30%) LMWH: (28%) Fonda: (42%) | 3 |
| Turrian (2022) | Retrospective Single center | Hemat (33%), Solid malign (64%)  Brain (3%) | 60 | 60,  Symptomatic | LMWH ( 90%)  Treatment unspecfied (10%) | Mean 18 |
| Hakem (2023) | Retrospective Single center | Hemat (32%), Solid malign (47%)  Others (17%)  Unspecified malig (3%) | 200 | 200,  Symptomatic 161;  asymptomatic 39 | LMWH (77%), DOAC (16%), warfarin (3%) | Median 16.5 mo (range, 10-36) |
| Xu (2023) | Retrospective Single center | Hematologic (18%)  Solid malign (55%) | 217 | 217,  - | Rivaroxaban (46%)  LMWH (54%) | Mean 3 |
| Ngo et al  (2024) | Retrospective Single center | Solid malign (100%) | 198 | 198,  - | DOAC/LMWH/VKA/UFH +/- line removal vs no AC +/- line removal | 12 |

Supplementary Table 4. Study characteristics. AC= anticoagulation, Solid malign = solid malignancies, Hemat = Hematological malignancies, GI = gastrointestinal LMWH = low molecular weight heparin, UFH: unfractionated heparin, VKA = vitamin K antagonist, DOAC; direct oral anticoagulant. Missing data is indicated with ‘-’.
